# Therapeutic Effects of Curcumin—From Traditional Past to Present and Future Clinical Applications

**DOI:** 10.3390/ijms20153757

**Published:** 2019-08-01

**Authors:** Beatrice E. Bachmeier, Dieter Melchart

**Affiliations:** 1 Competence Center for Complementary Medicine and Naturopathy [CoCoNat], Technical University Munich, 80807 Munich, Germany; 2 Institute of Laboratory Medicine, University Hospital, LMU Munich, 80807 Munich, Germany; 3 Institute for Complementary and Integrative Medicine, University Hospital Zurich and University of Zurich, CH-8091 Zurich, Switzerland

## Abstract

The efficacy of the plant-derived polyphenol curcumin, in various aspects of health and wellbeing, are a matter of public interest. An internet search of the term “Curcumin” displays about 12 million hits. Among the multitudinous information presented on partly doubtful websites, there are reports attracting the reader with promises ranging from eternal youth to cures for incurable diseases. Unfortunately, many of these reports are not based on scientific evidence, but they feed the desideratum of the reader for a “miracle cure”. This circumstance makes it very difficult for researchers, whose work is scientifically sound and evidence is based on the therapeutic benefits (or side effects) of curcumin, to demarcate their results from sensational reports that circulate in the web and in other media. This is only one of many obstacles making it difficult to pave curcumin’s way into clinical application; others are its nonpatentability and low economic usability. A further impediment comes from scientists who never worked with curcumin or any other natural plant-derived compound in their own labs. They have never tested these compounds in any scientific assay, neither in vitro nor in vivo; however, they claim, in a sometimes polemic manner, that everything that has so far been published on curcumin’s molecular effects is based on artefacts. The here presented Special Issue comprises a collection of five scientifically sound articles and nine reviews reporting on the therapeutic benefits and the molecular mechanisms of curcumin or of chemically modified curcumin in various diseases ranging from malignant tumors to chronic diseases, microbial infection, and even neurodegenerative diseases. The excellent results of the scientific projects that underlie the five original papers give reason to hope that curcumin will be part of novel treatment strategies in the near future—either as monotherapy or in combination with other drugs or therapeutic applications.

## 1. Curcumin’s Therapeutic Potential and Novel Therapeutic Approaches

The natural polyphenol curcumin is derived from the plant *Curcuma longa* Linn, a member of the Zingiberaceae, naturally occurring throughout tropical and subtropical regions of the world. Curcumin has been used in Ayurveda and traditional Chinese medicine for thousands of years to treat inflammatory diseases and bacterial infections [1]. 

### 1.1. Neoplastic Diseases

Because of its anti-apoptotic and antiproliferative efficacy, its ability to interfere with several tumor progression associated signaling pathways, and to modulate tumor-associated miRNA expression, curcumin is regarded as antitumorigenic [2,3]. In addition, curcumin prevents formation of breast and prostate metastases in vivo [4,5,6]. The review by Willenbacher et al. in this issue summarizes some papers that have been published in the field of curcumin’s antitumorigenic effects.

Curcumin is also potent against cancer types that are difficult to treat, like melanoma [7,8,9] or glioblastoma [10], as demonstrated by the work of Maiti et al. in this issue. They observed increased levels of autophagy and decreased levels of mitophagy markers, along with inhibition of the PI3K-Akt/mTOR pathway after treatment of glioblastoma cells with curcumin or solid lipid curcumin particles. Renal cell carcinoma is relatively rare, with rates of approximately 3% of all adult cancer patients; however, one-third of the patients have metastases at diagnosis and are resistant to most treatments like chemotherapy or radiation. In this context, complementary and alternative treatment strategies are highly desiderated by concerned patients. Blaheta and colleagues as well as Zöller and coworkers describe the very promising effects of a novel therapy combining the application of curcumin with visible light exposure. In detail, the combination therapy inhibits growth and proliferation of tumor cells and induces apoptosis.

In the context of curcumin’s molecular modes of action, resulting in its antitumorigenic effects, it seems likely to draw some attention on its molecular structure. Tomeh and coworkers bring some light into this aspect by summarizing what is so far known on the correlation between molecular mechanisms, cellular pathways, and structural characteristics of curcumin and its derivatives.

### 1.2. Aging

The ability to modulate the transcription factor NFκB explains curcumin’s anti-inflammatory effect [1,2,11,12,13]. Aging and age-related diseases also come along with chronic inflammation. The correlation between cancer and inflammation dates back to Virchow who suggested that “lymphoreticular” infiltrates reflect the origin of cancer at sites of chronic inflammation, and that there are striking similarities between ulcers, wound healing, and cancer [14]. The paper by Kujundži´ et al gives an overview on scientific data that would enable establishing connections and functional links between the specific of “inflamm-agin” and the cancer cell’s metabolism, its proliferative potential, and curcumin’s pleiotropic activity. In this context, Bielak-Zmijewska and coworkers also summarize scientific data on curcumin’s ability to postpone progression of age-related diseases in which cellular senescence is directly involved. They furthermore point out that curcumin causes elongation of the lifespan of model organisms and alleviates aging symptoms. In addition, they discuss thoroughly curcumin’s ability to modulate cellular senescence.

### 1.3. Inflammatory Disorders

Because of its scientifically evidenced characteristics to interfere with a variety of signal transduction pathways, transcription factors, and cellular processes, curcumin can potentially be applied in the treatment of many diseases (inflammatory disorders in particular). In this context, curcumin has been used to treat gastrointestinal diseases such as indigestion, flatulence diarrhea, and even gastric and duodenal ulcers [11,15,16]. Kwiecien and colleagues summarize in their review curcumin’s protective effects against esophageal and gastric disorders. In addition, curcumin is potentially efficacious against intestinal inflammatory diseases. Burge and colleagues discuss the beneficial effects of curcumin on the microbiome, its antimicrobial properties, inhibition of TLR4/NFκB/AP-1 signal transduction, changes in cytokine profiles, and alterations to immune cell maturation and differentiation. The combination of all these molecular actions makes curcumin a promising candidate to treat intestinal inflammatory diseases like necrotizing enterocolitis, Crohn’s disease, and ulcerative colitis.

Curcumin can also improve wound healing. Barchitta and coworkers point out that curcumin induces apoptosis of inflammatory cells during the early phase of wound healing and could accelerate the healing process by shortening the inflammatory phase. Moreover, curcumin might facilitate collagen synthesis, fibroblast migration, and differentiation.

### 1.4. Neurodegenerative Diseases

Lately, evidence has accumulated that curcumin has neuroprotective properties and is a candidate for the treatment of Alzheimer’s disease. In their review, Pluta and colleagues focus on the role and mechanisms of curcumin in inhibiting ischemia/reperfusion brain injury and potential therapeutic strategies in the treatment of ischemic brain damage of the Alzheimer’s disease phenotype. Comparably, Ferreira and colleagues also delineate neuroprotective characteristics by summarizing what is known about the role of curcumin on transthyretin amyloidosis. According to previous reports, curcumin modulates abnormal transthyretin (TTR) aggregation and inhibits its deposition in the tissue. The pleiotropic activities of curcumin provide multiple ways to tackle TTR pathophysiology, through direct interaction of curcumin with TTR, or indirect effects affecting signaling pathways associated with TTR amyloid fibril formation and clearance.

### 1.5. Infectious Diseases

The treatment of bacterial infections has become extremely challenging due to resistance against antibiotics available in the pharmaceutical market. Moreover, another important issue to be addressed is that common antibiotics evoke adverse events. In this context, phytotherapeutic approaches become popular for patients who are searching for alternatives to standard treatments. There are numerous reports that have already delineated not only the antibacterial but also the antiviral and antifungal activities of curcumin. [17]. In this Special Issue, Czernicka and coworkers focus on the antimicrobial potential of single components of the *Curcuma longa* crude extract against a variety of Gram-positive bacteria strains.

### 1.6. Remarks on Solubility and Bioavailability

Despite curcumin’s therapeutic potential in vitro and in vivo, it has to be considered that the molecule is lipophilic and hardly soluble in water. Up to now it is not fully understood how curcumin reaches the target organ in order to exert its therapeutic effects and how it becomes metabolized in the human body. In order to overcome these pharmacological problems, several attempts have been undertaken to encapsulate curcumin into nanoparticles in order to ensure that curcumin is transported easily in the bloodstream. In the current issue, Kong and coworkers present their data on a novel formulation of curcumin-loaded mesoporous silica nanoparticles with higher antioxidant activity, antitumor activity, higher cytotoxicity, and stability as compared to the curcumin molecule itself. However, cytotoxicity of these nanoparticle carriers has to be explored in depth before we get too enthusiastic about this idea.

## 2. Conclusions

In recent years, curcumin’s reputation regarding its therapeutic effects has been damaged. In molecular drug screening tests, and partly polemic publications, curcumin has been declared to belong to the PAINS (pan assay interference compounds) [18] and to yield confusing results because curcumin does not have one single drug target [19]. The authors of these disparagements gained their information from results generated in high-throughput screenings. However, high-throughput screenings are prone to technical artefacts and, therefore, are a deceptive tool because potential drug candidates could be missed. Additionally, the fact that curcumin, like many other natural compounds, has more than one drug target speaks to its versatile applicability and its low risk to cause acquired therapy resistance [20,21,22].

A crucial state of mind is prerequisite for good quality of research and reputable scientist questions not only the results of other fellow colleagues but also the own. It is our task and responsibility to provide high-quality research, and it can happen, of course, that carelessly executed research projects and results are published, sometimes even in high-ranked papers. 

However, this holds true not only for curcumin but also for other bioactive compounds, no matter if they are plant-derived or if they have been developed and chemically synthesized in the pharma industry. While reasonable doubt is essential for sound, scientific results, it makes no sense to disparage everything that has so far been published on curcumin’s therapeutic effects in treating chronic and neoplastic diseases and to declare that all results are artefacts. Instead, we should accept the challenge to distinguish between scientifically sound and false results; otherwise, we lose a promising candidate for complementary and alternative treatment strategies. 

Additionally, we should not challenge treatment successes of Ayurveda or traditional Chinese medicine, where plant-derived compounds like curcumin have been applied efficaciously to treat inflammatory diseases and bacterial infections for thousands of years. To disclaim treatment successes of traditional medicine would simply be ignorant.

Curcumin’s detractors criticize that it has never been shown to be conclusively effective in a randomized, placebo-controlled clinical trial for any indication [19]. To this end it has to be considered that it is almost impossible to get financial support to conduct a clinical trial with a substance that cannot be patented and, therefore, is economically uninteresting. Another point is the study design—curcumin cannot be tested in randomized, placebo-controlled trials because, nowadays, clinical trials are performed in the form “study compound against standard therapy”, otherwise the trial would not get a positive vote from the ethics committee. Therefore, the question is: against which compound should we test curcumin? 

There is no doubt, that due to the comprehensive data from preclinical studies, together with first results from single patients or small cohorts, the next task on the list has to be to test Curcumin in well-designed clinical trials. However, the greatest challenge will be to find sponsors for clinical research on curcumin, as this promising plant-derived compound cannot be exploited economically.
